# *Gbx1* and *Gbx2* Are Essential for Normal Patterning and Development of Interneurons and Motor Neurons in the Embryonic Spinal Cord

**DOI:** 10.3390/jdb8020009

**Published:** 2020-04-01

**Authors:** Desirè M. Buckley, Jessica Burroughs-Garcia, Sonja Kriks, Mark Lewandoski, Samuel T. Waters

**Affiliations:** 1 Department of Molecular Biosciences, University of Texas at Austin, Austin, TX 78712, USA; desirembuckley@gmail.com; 2 Section of Pediatric Hematology-Oncology, Department of Pediatrics, University of Oklahoma Health Sciences Center, Oklahoma City, OK 73104, USA; jib6x6@gmail.com; 3 Neurona Therapeutics, South San Francisco, CA 94080, USA; sonja@neuronatx.com; 4 Cancer and Developmental Biology Laboratory, National Cancer Institute, Frederick, MD 21702, USA; lewandom@mail.nih.gov; 5 Division of Sciences and Mathematics, University of the District of Columbia, Washington, DC 20008, USA

**Keywords:** *Gbx1*, *Gbx2*, spinal cord, mouse, development

## Abstract

The molecular mechanisms regulating neurogenesis involve the control of gene expression by transcription factors. *Gbx1* and *Gbx2*, two members of the Gbx family of homeodomain-containing transcription factors, are known for their essential roles in central nervous system development. The expression domains of mouse *Gbx1* and *Gbx2* include regions of the forebrain, anterior hindbrain, and spinal cord. In the spinal cord, *Gbx1* and *Gbx2* are expressed in PAX2^+^ interneurons of the dorsal horn and ventral motor neuron progenitors. Based on their shared domains of expression and instances of overlap, we investigated the functional relationship between *Gbx* family members in the developing spinal cord using *Gbx1*^−/−^, *Gbx2*^−/−^, and *Gbx1*^−/−^/*Gbx2*^−/−^ embryos. In situ hybridization analyses of embryonic spinal cords show upregulation of *Gbx2* expression in *Gbx1*^−/−^ embryos and upregulation of *Gbx1* expression in *Gbx2*^−/−^ embryos. Additionally, our data demonstrate that *Gbx* genes regulate development of a subset of PAX2^+^ dorsal inhibitory interneurons. While we observe no difference in overall proliferative status of the developing ependymal layer, expansion of proliferative cells into the anatomically defined mantle zone occurs in *Gbx* mutants. Lastly, our data shows a marked increase in apoptotic cell death in the ventral spinal cord of *Gbx* mutants during mid-embryonic stages. While our studies reveal that both members of the *Gbx* gene family are involved in development of subsets of PAX2^+^ dorsal interneurons and survival of ventral motor neurons, *Gbx1* and *Gbx2* are not sufficient to genetically compensate for the loss of one another. Thus, our studies provide novel insight to the relationship harbored between *Gbx1* and *Gbx2* in spinal cord development.

## 1. Introduction

The dorsal and ventral regions of the spinal cord are functionally and anatomically distinct, interacting through complex neuronal circuits. Assembly of neuronal circuits in the spinal cord depends on specification and patterning of distinct neurons in the dorsal and ventral horns [1,2]. Moreover, it has become clear that many essential aspects of the developmental programing are directed by restricted expression profiles of transcription factors in a cell type specific manner [3,4,5,6]. 

Dorsal spinal interneurons receive, process, and modulate sensory information from the periphery associated with touch, pain, and body position. The information is then relayed to somatosensory centers in the brain and motor neurons in the ventral spinal cord [4,7,8]. Neurogenesis in the dorsal spinal cord occurs in two distinct stages and is mediated by the combinatorial expression of basic helix-loop-helix (bHLH) and homeodomain (HD)-containing transcription factors along the DV axis. Six classes of neural progenitors, which give rise to post-mitotic interneurons (dI1–dI6), are generated during the first stage, embryonic day (E) 10–E11.5 and populate the deep layers of the dorsal horn [5,6,9]. Differentiation of dorsal progenitor cells into post-mitotic inhibitory or excitatory interneurons is regulated by the restricted and transient expression of HD proteins [10,11]. Of the three most ventral classes of dorsal interneurons, dI4 (*Gsh1/2*^+^, *Lbx1*^+^, *Ptf1a*^+^, *Pax2*^+^) are inhibitory whereas, dI5 (*Gsh1/2*^+^, *Lbx1*^+^, *Ptf1a*^−^, *Tlx1/3*^+^) are excitatory [11,12,13]. Further changes in transcription factor expression profiles in a subset of (*Gsh1/2*^+^, *Ascl1*^+^) progenitor cells, occurs during the second stage of neurogenesis (E12–E14.5), resulting in the generation of a common pool of late-born dIL progenitors and subsequently dILA (*Lbx1*^+^, *Ptf1a*^+^, *Pax2*^+^) inhibitory GABAergic and dILB (*Lbx1*^+^, *Ptf1a*^−^, *Tlx1/3*^+^) excitatory glutamatergic interneurons. Most of the superficial dorsal horn, substantia gelatinosa (lamina II), and lamina propria (laminae III–IV), is comprised of dIL-derived neurons [4,5,12,14]. 

The competence of spinal neural circuits is not only dependent on the molecular composition of neurons, but also quantity of cells generated during development. This balance is coordinated by the proliferative capacity of neural precursor cells to divide and/or differentiate. In vertebrates, cell proliferation occurs within the ventricular zone (VZ) of the spinal cord (Smart IH 1972). Regulation of this event is the function of several morphogenic signaling families, including FGFs and Wnts reviewed in [15]. For dorsal neural progenitors, the early-born proliferative cells (E10–E11.5) give rise to dI4 and dI5 interneurons, while the later-born class (E12–E14.5) generates dILA and dILB interneurons [5,6].

In contrast to dorsal interneurons, ventral spinal cord interneurons modulate and direct motor control [16,17]. Activity of bHLH and HD-containing transcription factors also regulates the specification of five ventral progenitor domains along the dorso-ventral (DV) axis, progenitor motor neurons (pMN), and (pV0–pV3), which give rise to molecularly distinct populations of motor neurons and ventral interneurons. Similar to dorsal interneurons, differentiation of ventral progenitor cells into post-mitotic inhibitory and excitatory interneurons is regulated by the restricted and transient expression of HD proteins [1,2,18].

One class of homeobox genes, gastrulation brain homeobox (*Gbx*), has been shown through recent studies, as essential for correct patterning and maintenance of neurons and motor neurons along the anteroposterior (AP) axis of the developing neural tube [19,20,21,22,23,24,25,26,27,28]. *Gbx* genes encode two closely related and evolutionarily conserved HD-containing transcription factors, GBX1 and GBX2. A defining feature of the Gbx family is a highly conserved 60 amino acid DNA-binding domain, which differs by just three amino acids [29]. In addition to having highly conserved functional domains, *Gbx1* and *Gbx2* expression domains overlap in several areas of the developing central nervous system (CNS) including the forebrain, anterior hindbrain, and spinal cord. 

In the mouse spinal cord, *Gbx2* is heavily expressed in the dorsal ventricular and mantle zones and two ventral lateral stripes at E10.5. Expression of *Gbx2* in the dorsal mantle zones persists through E12.5. However, *Gbx2* mRNA expression in the spinal cord is rapidly downregulated between E12.5 and E13.5 [30]. Recent fate mapping studies in mouse embryos have also demonstrated that a subset of ISL1^+^ ventral motor neurons, as well as PAX2^+^ dorsal and ventral interneurons of the spinal cord are derived from the *Gbx2* lineage [21]. Moreover, these studies revealed a decrease in mitotically active cells in the dorsal half of the neural tube of *Gbx2*^−/−^ embryos at E10.5, while concomitantly showing overt mispatterning to several dorsal interneurons populations, suggesting similar function of *Gbx2* in the anterior hindbrain and spinal cord [19,24].

Mouse *Gbx1* expression is detected within the neural plate by E8.25. By E9.0, *Gbx1* mRNA transcripts are broadly detected within the ventricular zone of the developing spinal cord, and become restricted to the dorsal mantle zones by E12.5 [29,30]. GBX1 protein expression in the dorsal mantle zones colocalizes with a subset of (LBX1^+^, LHX1/5^+^, PAX2^+^) GABAergic interneurons during the second stage (E12–E14.5) of dorsal neurogenesis and persists through early postnatal stages [26,28,30]. More recent molecular analyses of mouse embryos homozygous for a *Gbx1* null allele revealed disrupted assembly of the proprioceptive sensorimotor circuit within the spinal cord, and a marked reduction of ISL1^+^ ventral motor neurons beginning at E14.5 [25].

Interestingly, there is accumulating evidence demonstrating that many closely related DNA-binding transcription factors with overlapping expression domains are genetically redundant and able to functionally compensate for family members having reduced levels of expression due to hypomorphic or inactivation mutations [31,32,33,34]. This notion was recently demonstrated for the *Gbx* gene family in the cerebellar primordium where they are redundantly functional for morphogenesis and subsequent differentiation [27]. Hence, the transient, partially overlapping expression domains of *Gbx1* and *Gbx2* in the developing spinal cord raises the possibility that genetic and/or functional redundancy between *Gbx* genes may occur. Both GBX proteins colocalize with PAX2^+^ interneurons in the dorsal mantle zones beginning at E12.5 [21,26,28,30]. In the ventral spinal cord, *Gbx1* and *Gbx2* are expressed in subsets of pMNs. Importantly, *Gbx* loss-of-function mutants display a marked depletion of subsets of ISL1^+^ motor neurons [21,25]. However, similar to homologs of many transcription factor families, homozygous inactivation of *Gbx1* and *Gbx2* in mice, reveal very different consequences. 

Key to understanding the genetic relationship between *Gbx1* and *Gbx2* in development of distinct spinal neurons is determining the relative functional properties of each gene individually. Here, we investigate possible genetic and functional redundancy between *Gbx1* and *Gbx2* during spinal cord development by examining the impact of their functional loss on late-born PAX2^+^ dorsal interneurons and ISL1^+^ motor neurons in single *Gbx1*^−/−^, *Gbx2*^−/−^ mutant embryos. We also created a double (d) *Gbx1*^−/−^/*Gbx2*^−/−^ knockout (KO) embryo, to facilitate our genetic interaction analyses. Our data indicate that deletion of either family member results in upregulation of its counterpart in the dorsal mantle zone. Interestingly, our results show abnormal patterning of proliferating cells within the dorsal VZ, and significant decrease in a subset of dorsal PAX2^+^ interneurons in single and dKO *Gbx* mutants. In the ventral spinal cord, we observed a striking increase in apoptotic cell death of motor neurons in single *Gbx* mutants, which is augmented in dKO mutants. Taken together, our studies demonstrate that *Gbx* genes do not subserve redundant functionality in the spinal cord as shown in the anterior hindbrain. Rather, our data reveals a stringent relationship between *Gbx1* and *Gbx2* in patterning a subset of dorsal interneurons and ventral motor neurons, providing new insights into their molecular function in spinal cord development.

## 2. Materials and Methods

### 2.1. Generation of Gbx1^−/−^/Gbx2^−/−^ Mice

Mice carrying the mutant null allele for the *Gbx1* gene (*Gbx1*^−/−^) were generated through cre-mediated excision of exon 2, which contains the functional DNA-binding homeodomain [25]. *Gbx1*^−/−^ mice are viable and reproductively competent. Mice carrying the mutant null allele for the *Gbx2* gene (*Gbx2*^−/−^) were also generated through cre-mediated excision of the homeobox contained in exon 2 [20]. However, *Gbx2*^−/−^ mutant embryos die the day of birth. Thus, mutants carrying a homozygous allele for the *Gbx2* gene are obtained by the mating of mice heterozygous for the *Gbx2* null allele, which are viable and phenotypically indistinguishable from wildtype littermates. In this study, embryos carrying null alleles for both *Gbx* genes (*Gbx1*^−/−^/*Gbx2*^−/−^) are obtained by mating parental lines composed of genetic combinations of *Gbx1*^−/−^/*Gbx2*^−/+^ or *Gbx1*^−/+^/*bx2*^−/+^. The primers for genotyping all mutant animals are identical to those described in [19,20]. Timed pregnant mice were used for all experiments. At noon of the day the vaginal plug that was observed was considered E 0.5. 

### 2.2. Immunohistochemistry

For immunohistochemistry analyses, *Gbx1*^−/−^, *Gbx2*^−/−^, *Gbx1*^−/−^/*Gbx2*^−/−^ and control embryos were dissected and subsequently fixed with 4% paraformaldehyde (PFA) in 1X phosphate-buffered saline (PBS) for 2 h at 4 °C, washed three times in 1X PBS for 1 h, equilibrated with 25% sucrose overnight at 4 °C and embedded in optimal temperature tissue (OCT) (tissue-tec) for cryosectioning. Transverse, serial 12 µm cryosections were made along the length of the lumbar-sacral spinal cord. 

### 2.3. Islet1 and PAX2

Sections were washed with 0.1% Triton X-100 in 1X PBS (PBST), blocked with 1X PBS containing 10% lamb serum, 1% bovine serum albumin, and 0.25% Triton X-100 for 90 min and incubated with the appropriate primary antibodies diluted in blocking solution at 4 °C overnight. The following day, sections are washed briefly with PBST and incubated with the appropriate fluorescently-conjugated secondary antibodies diluted in blocking solution at 4 °C overnight. The following primary and secondary antibodies were used at the given dilution: Mouse monoclonal mouse anti-Islet1 (1:100, 39.45D-DSHB), rabbit anti-PAX2 (1 mg/mL, Invitrogen), goat antirabbit AlexaFluor 488 (1:500; Invitrogen), and goat antimouse AlexaFluor 568 (1:500; Invitrogen, Carlsbad, CA, USA). Stained sections were dehydrated in serial dilutions of ethanol in 1X PBS and mounted using DPX mounting media. 

### 2.4. Caspase-3 Staining and Phosphorylated Histone H3

For the labeling of apoptotic cells expressing caspase-3 or mitotically active cells expressing phosphorylated histone H3 (H3P), sections were post-fixed with 3% formaldehyde in 1X PBS (caspase-3) or 10% neutral buffered formalin (H3P), washed twice with 1X PBS, incubated with 3% (*w*/*v*) hydrogen peroxidase in methanol to quench endogenous peroxidase activity, washed twice with 1X PBS, blocked with 1X PBS containing 0.3% Triton-X 100 and 5% normal goat serum for 1 h at RT, followed by an overnight incubation at 4 °C with rabbit α-caspase-3 (cell signaling, 1:1500) or rabbit α-phospho-H3 (Millipore; 1:500) primary antibody in blocking solution. Sections are then washed three times with 1X PBS, and incubated overnight at 4 °C with biotinylated α-rabbit IgG secondary antibody (Vector Laboratories #PK-6101) diluted in blocking solution. To visualize localization of the antigen, sections are washed twice with 1X PBS, incubated with VECTASTAIN Elite ABC (Vector Laboratories #PK-6101) reagent for 30 min at RT, incubated with 1X PBS containing 1% DMSO six times for 15 min at RT, then incubated with 0.5 mg/mL 3,3′-diaminobenzidine tetrahydrochloride (DAB #D9015; Sigma-Aldrich, St. Louis, MO, USA) diluted in 1X PBS containing 1% DMSO for 15 min at RT. Peroxidase reaction is initiated by the addition of 200 µL of 0.0003% H_2_O_2_ to 1X PBS containing 0.5 mg/mL DAB and 1% DMSO. The reaction is stopped by washing sections several times with ice cold 1X PBS. Slides are mounted with 70% glycerol/1X PBS.

### 2.5. Section In Situ Hybridization

For section RNA in situ hybridizations analyses, *Gbx1*^−/−^, *Gbx2*^−/−^, *Gbx1*^−^^−/−^/*Gbx2*^−/−^ and control embryos were dissected and subsequently fixed with 4% paraformaldehyde (PFA) in 1X PBS for 2 h at 4 °C, washed three times in 1X PBS for 1 h, equilibrated with 25% sucrose overnight at 4 °C and embedded in optimal temperature tissue (OCT) (tissue-tec) for cryosectioning. Transverse, serial 12 µm cryosections were made along the length of the lumbar-sacral spinal cord. In situ hybridizations were performed using digoxigenin (Roche Molecular Biochemicals) labeled probes specific for mouse *Gbx2* and *Gbx1*, as described elsewhere [29].

### 2.6. Microscopy

Analysis of immunostained spinal cords sections were examined and photographed using the Leica DM5500 under 10×, 20×, and 40× ocular magnification. In situ hybridization images were captured using the Leica DFC290 camera. Identical parameters were used consistently for each sample within an experiment during imaging. 

### 2.7. Statistical Analysis

PAX2, H3P, and caspase-3 cell counting was performed on three lumbar spinal cord sections from three embryos of each genotype analyzed (i.e., nine sections each for control, *Gbx1*^−/−^, *Gbx2*^−/−^, *Gbx1*^−/−^/*Gbx2*^−/−^). For PAX2, positive cells within a single quadrant of the superficial dorsal horn were counted for the left and right side of the spinal cord for each section to minimize counting errors. For H3P, positive cells within a single quadrant of the dorsal ependymal layer were counted to assess the level of proliferation within the ventricular zone. Additionally, H3P-positive cells within a single quadrant of the dorsal mantle zone were counted to assess the level of proliferation outside of the ventricular zone. For caspase-3, cells positively marked by the antigen within a quadrant aligned to the ventral-most boundary of the ventral spinal cord were counted. Left and right sides were quantified to minimize counting errors. Statistical differences in cell numbers were calculated using one-way ANOVA. Statistically significant results were further assessed using Tukey’s honestly significant difference (HSD) post-hoc test to determine which group’s means (compared with each other) differ (Graphpad Prism software). Expression of *Gbx1* or *Gbx2* mRNA in the dorsal horn was quantified using ImageJ. Statistical differences in signal intensity were assessed using Student’s t-test (Graphpad Prism software). Results are represented as mean ± SEM, and samples are considered statistically significant by having a value of *p < 0.05, ** *p* < 0.01, *** *p* < 0.001, **** *p* < 0.0001.

### 2.8. Animal Ethics Statement

Animal experimentation protocols were reviewed and approved by the University of Missouri IACUC (protocol #7561).

## 3. Results

### 3.1. Increase in Embryonic Spinal Cord Expression of Gbx1 and Gbx2 in Homozygous Null Counterparts 

**Figure 1:**
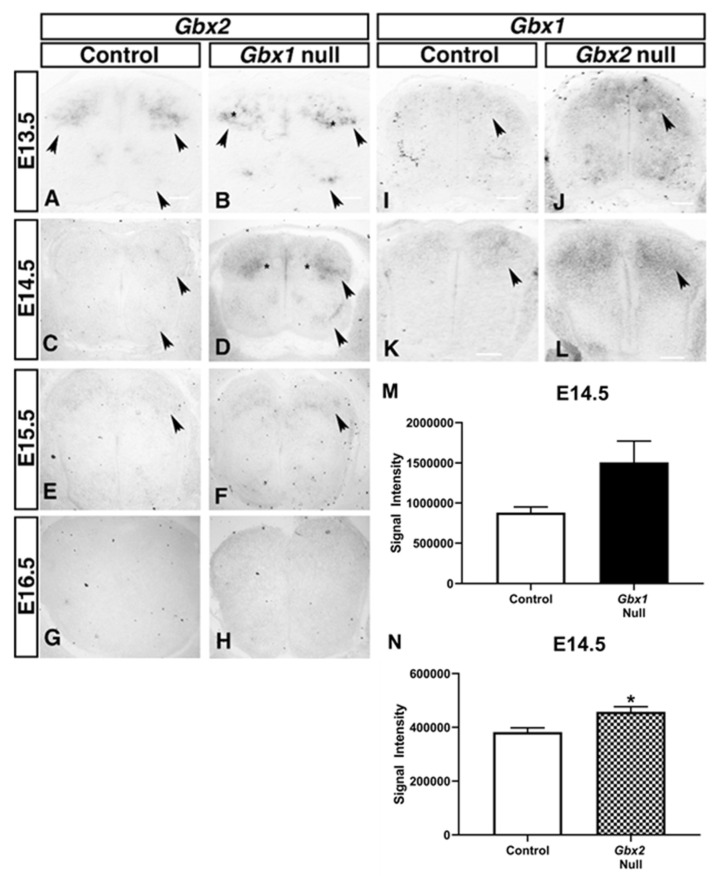
*Gbx* mRNA expression is transiently upregulated in null counterparts during midembryonic stages. *Gbx2* and *Gbx1* mRNA expression analysis between embryonic day (E)13.5–E16.5 in *Gbx2*^−/−^, *Gbx1*^−/−^, and age-matched control, lumbar spinal cord sections. In control embryos at E13.5, *Gbx2* is expressed within neural populations of the developing dorsal mantle zone (**A**; black arrowhead). *Gbx2* expression pattern remains consistent in *Gbx1*^−/−^ at E13.5, however, *Gbx2* is expressed to a greater extent throughout laminae (III–IV) (**B**; asterisk). Persistent upregulation of *Gbx2* expression in *Gbx1*^−/−^ spinal cord occurs through E14.5, when compared to control embryo (compare **D** with **C**; black asterisk). *Gbx2* expression is barely observable in the control embryo at E15.5 (**E**), and this intensity is comparable in the mutant spinal cord (**F**), which marks the stage in which upregulation of *Gbx2* terminates. By E16.5, *Gbx2* expression is absent from the spinal cord of control and mutant embryos (**G**–**H**, black arrowhead). *Gbx1* expression pattern recapitulates that of the control embryo at E13.5 (**I**), however, *Gbx1* is expressed more broadly and intensely throughout the dorsal horn in *Gbx2*^−/−^ mutants (**J**). A significant increase in *Gbx1* expression persists in the spinal cord of *Gbx2*^−/−^ mutants through E14.5, when compared to control embryos (**K**–**N**). Scale bars represent 100 µm. N = 3 for each genotype, at each stage. Data in graphs shown as mean + SEM. Asterisk indicates significant difference (* *p* < 0.05, Student’s t-test).

A significant role for *Gbx* transcription factors in neural development has been established. However, while their mRNA expression in the spinal cord overlap spatially and temporally, they are not biochemically identical. Whereas both *Gbx1* and *Gbx2* are heavily expressed in the dorsal mantle zone at E12.5, *Gbx2* expression diminishes after E13.5 [30] (Figure 1), while *Gbx1* expression persists through postnatal stages. Key in understanding phenotypic differences and similarities in *Gbx* homozygous null mutants and relative functions of *Gbx1* and *Gbx2* in spinal cord development [19,20,25,35,36], is whether the observed loss-of-function phenotypes are determined by differences in spatial and temporal expression or biochemical differences between the proteins.

We have previously shown that *Gbx1* expression is not overtly upregulated outside the CNS in the absence of *Gbx2* [29]. In addition, we and others, have shown that *Gbx2* is robustly expressed in the dorsal mantle zones of wildtype control embryos and is then rapidly downregulated beginning at E12.5 [29,30]. Therefore, to examine if any genetic interaction occurs between *Gbx* genes, we first sought to determine if the loss of *Gbx1* impacts expression of *Gbx2* in the spinal cord. We observed a striking increase of *Gbx2* expression in the dorsal mantle zones of *Gbx1*^−/−^ embryos when compared to wildtype control embryos at E13.5 (Figure 1A,B) [28]. We observed increased levels of *Gbx2* mRNA throughout the superficial dorsal horn of *Gbx1*^−/−^ embryos. The predominant expression appears localized in laminae (III–IV) (Figure 1, asterisk). Further examination at later developmental stages, E14.5–E16.5 (Figure 1C–H), demonstrates that elevated levels of *Gbx2* expression persist in the dorsal spinal cord of *Gbx1*^−/−^ embryos at E14.5 and E15.5 (Figure 1D,F). However, *Gbx2* expression in *Gbx1* mutants is nearly absent at E15.5. The apparent qualitative increase of *Gbx2* mRNA is consistent with our assessment of *Gbx2* expression in *Gbx1* mutants using quantitative real-time PCR, revealing a marked increase of *Gbx2* transcripts at E13.5 [37]. 

Next, we examined if *Gbx1* expression is altered in *Gbx2*^−/−^ spinal cords. We analyzed *Gbx1* expression by in situ analysis in control and *Gbx2*^−/−^ spinal cords at E13.5 and E14.5 (Figure 1I–L). Our results reveal a significant increase of *Gbx1* expression in the dorsal spinal cord of its null counterpart at E14.5 (*p* = 0.0238) (Figure 1L,N). Taken together, these observations show that loss-of-function of one *Gbx* family member invokes higher levels of expression in its counterpart. 

**Figure 2:**
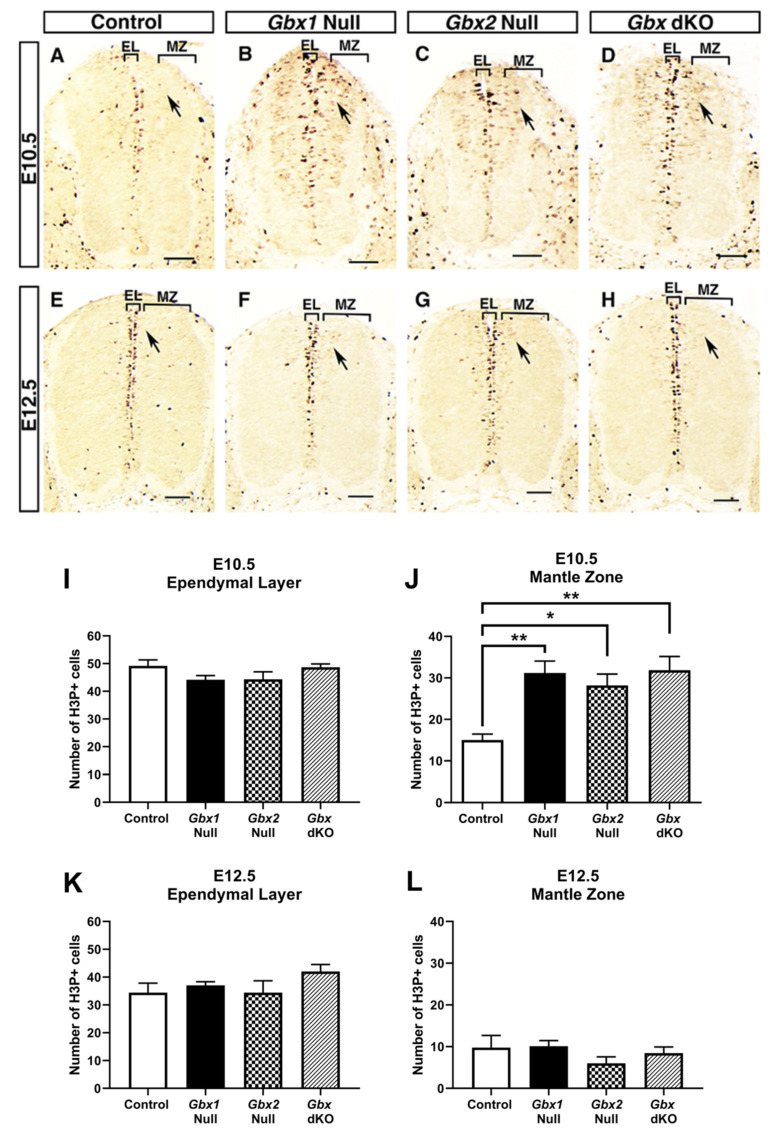
Absence of *Gbx* gene expression results in aberrant patterning of proliferative cells in the dorsal spinal cord of *Gbx* mutants. Immunolabeling of apoptotic cells with phosphorylated histone H3 (H3P) at E10.5 (**A**–**D**) and E12.5 (**E**–**H**). At E10.5, during the earliest stages of spinal neuron proliferation, we observe a striking expansion of H3P^+^ cells in *Gbx* mutants (compare **B**–**D** with **A**). While the number of proliferative cells, correlating with the level of proliferation, remains consistent within the ependymal layer (EL), the number of labeled cells increased significantly within the mantle zone (MZ) at E10.5 (black arrowhead). Quantification of H3P^+^ cells within the EL (**I**) and MZ (**J**) reveal a significant increase in the number of cells within the MZ but not EL of all *Gbx* mutants at E10.5. Expansion of H3P^+^ cells is diminished in the EL and MZ by E12.5 (**K,L**). One-way ANOVA comparing the number of H3P^+^ cells within the MZ at E10.5 revealed an overall significant difference (F (3, 20) = 8.532, *p* = 0.0008). Post-hoc Tukey’s HSD revealed significant differences between all *Gbx* mutants compared to control. Results are shown as means ± SEM. Samples considered statistically significant have a value of * *p* < 0.05, ** *p* < 0.01. Scale bars represent 100 µm.

We next sought to examine the proliferative capacity of neural progenitors in spinal cords of control, *Gbx1*^−/−^, *Gbx2*^−/−^, and *Gbx* dKO littermate embryos. We have previously reported involvement of *Gbx2* in cell proliferation during cerebellar development. Immunohistochemistry was performed on transverse sections of lumbar segments of the spinal cord for each genotype, using an antibody specific for phosphorylated histone H3 (H3P), a marker for mitotically active cells [19]. We assessed the proliferative status of spinal cords at E10.5 and E12.5, the major developmental stages of neurogenesis for early-born and late-born classes of interneurons within the dorsal horn, respectively. Interestingly, we observed a striking difference, in which *Gbx* mutants appear to have an expanded zone of proliferation (Figure 2). Qualitative and quantitative analyses did not reveal a major difference in the uniformity or number of mitotically active cells constituting the ependymal/ventricular layer (EL) along the surface of the central canal between control and *Gbx* mutant spinal cords at either stage of development (Figure 2A–H). However, at E10.5, we observed a significant expansion of H3P^+^ cells outside the normal zone of proliferation as demonstrated in control embryos, into the mantle zone (MZ) (Figure 2B–D,J) (*p* < 0.05) in all *Gbx* mutants. However, expansion of H3P^+^ cells within the remaining psuedostratified epithelium of *Gbx* mutants is diminished by E12.5 (Figure 2 F–H,L) [38]. Similar to the anterior hindbrain of *Gbx2* mutants, these results strongly suggest that the proliferative capacity of dorsal neural progenitor cells is affected in *Gbx* mutants. 

### 3.2. Loss of Gbx1 and Gbx2 Results in Abnormal Development of PAX2^+^ Dorsal Spinal Cord Neurons 

**Figure 3:**
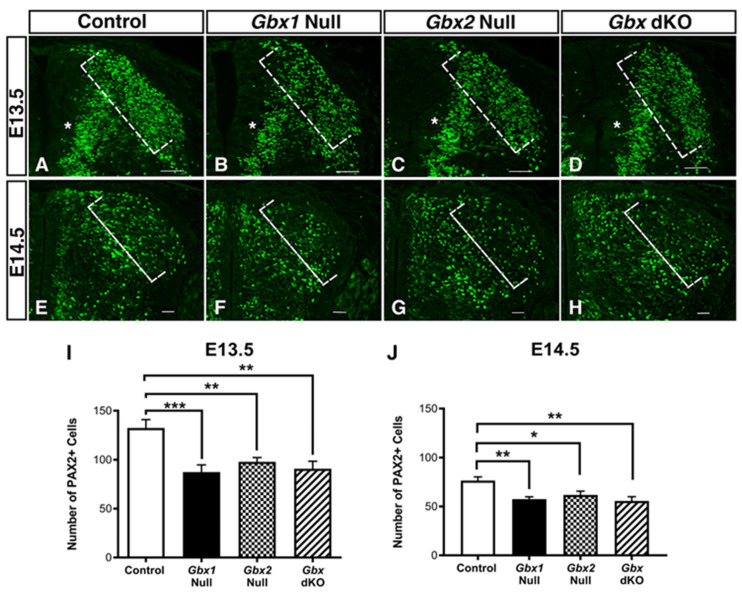
Loss of *Gbx1* and/or *Gbx2* expression results in reduction of PAX2^+^ cells in the superficial dorsal horn. Expression analysis of PAX2^+^, a marker of inhibitory interneurons at E13.5 (**A**–**D**) and E14.5 (**E**–**H**) in lumbar spinal cords of control, *Gbx1*^−/−^, *Gbx2*^−/−^, and *Gbx* double knockout (dKO) mutants. In control embryos at E13.5, the characteristic morphology of the dorsal spinal cord begins to emerge as interneurons populate the deep and superficial layers of the dorsal horn (**A**; white bracket). Proliferative progenitor cells of late-born class B interneurons within the deep dorsal horn (**A**; white asterisk). Analysis at E13.5 reveals loss of PAX2^+^ immunoreactivity in the most superficial layers of the dorsal horn in *Gbx1*^−/−^ (**B**); *Gbx2*^−/−^ (**C**), and *Gbx* dKO embryos (**D**), accompanied by additional loss in PAX2^+^ immunoreactivity within progenitor cells (**A**–**D**; white asterisk). One-way ANOVA comparing the number of PAX2^+^ cells in the lateral regions of the superficial dorsal laminae revealed an overall significant decrease (F (3,44) = 8, *p* = 0.0002). Post-hoc Tukey’s HSD revealed significant differences in PAX2^+^ cells for all *Gbx* mutants when compared to age-matched controls at E13.5 (**I**) and E14.5 (**J**). Results are shown as mean ± SEM. Samples considered statistically significant have a value of * *p* < 0.05, ** *p* < 0.01, *** *p* < 0.001. Scale bars represent 100 µm. (n = 3–5) for each genotype and embryonic stage examined.

*Gbx1* is expressed in a subset of late-born GABAergic interneurons of the developing dorsal horn, the class B dILA interneurons [30]. The *Gbx2* lineage has also been shown to contribute to PAX2^+^ inhibitory dILA interneurons, and maintenance of the proliferative status of dorsal progenitors [21]. Therefore, to investigate the role of the *Gbx* family in development of inhibitory interneurons in the dorsal spinal cord, we analyzed *Gbx1*^−/−^ and *Gbx2*^−/−^ embryos. Transverse, lumbar spinal cord sections from *Gbx1*^−/−^, *Gbx2*^−/−^, and littermate control embryos were assessed at E13.5 (Figure 3A–D) and E14.5 (Figure 3E–H) by immunohistochemistry examining PAX2^+^, the cell-type specific marker of dorsal inhibitory interneurons [5,11]. In control embryos at E13.5, PAX2^+^ cells span the medio-lateral axis of the dorsal spinal cord as they migrate into the various laminae (Figure 3A). At E13.5, the superficial layers of the dorsal horn are significantly less-densely populated with PAX2^+^ cells in *Gbx1*^−/−^ and *Gbx2*^−/−^ mutant spinal cords (*p* = 0.004 and *p* = 0.0081, respectively) (Figure 3B,C,I). By E14.5, PAX2^+^ expression within the most peripheral regions of the superficial laminae remains significantly reduced in both mutants, when compared to the age-matched control (*p* = 0.0037 and *p* = 0.0334, respectively) (Figure 3E–G,J). The apparent reduction of PAX2^+^ cells appears to span not only the peripheral, but also the medial regions of laminae II–IV (asterisk, Figure 3A–C), affecting the overall presence of PAX2^+^ expression in the dorsal spinal cord when compared to control. We further analyzed the observed differences in PAX2^+^ expressing cells between control embryos and *Gbx* single mutants quantitatively. Consistent with our qualitative assessment, quantification of PAX2^+^ cells reveals a significant decrease in the number of inhibitory interneurons within the superficial laminae of the dorsal horn in *Gbx1*^−/−^ and *Gbx2*^−/−^ mutants (Figure 3I,J). Moreover, our observed reduction of PAX2^+^ cells in *Gbx1*^−/−^ spinal cords is consistent with results from recent studies [28].

The observation that null mutations for either gene of the *Gbx* family results in the loss of PAX2^+^ cells within the superficial layers of the dorsal spinal cord, prompted us to examine the effects of inactivating both genes simultaneously on maintenance of PAX2^+^ in dorsal inhibitory interneurons. Transverse sections of the lumbar cords of *Gbx* dKO embryos were assessed in the same manner as the single mutants for comparison. At E13.5 and E14.5, *Gbx* dKO embryos display similar reduction in PAX2^+^ expression as in each of the single mutants, when compared to age-matched control embryos (Figure 3A,D). Quantification of cells immunopositive for PAX2^+^ in the superficial layers of *Gbx* dKO embryos at E13.5 and E14.5, confirms our qualitative observations as for each of the single *Gbx* mutants, and shows that there is a significant diminution in the number of PAX2^+^ cells (*p* = 0.0011; *p* = 0.0012, respectively) (Figure 3I,J). These data show that the inactivation of *Gbx* gene members individually, is sufficient to induce a significant loss of PAX2^+^ expression in neurons of the superficial dorsal spinal cord, and the simultaneous inactivation of both *Gbx* genes results in the same defect. Taken together these data suggest that members of the *Gbx* gene family share a functionally similar role in the development of late-born PAX2^+^ interneurons that settle into the most superficial layers of the dorsal spinal cord.

### 3.3. Loss of Gbx Transcription Factor Function Results in Increased Apoptosis in the Ventral Spinal Cord 

We next examined the impact of *Gbx* function on cell survival. Recent studies have provided intriguing evidence supporting a role for *Gbx* genes in motor neuron development. Results from our studies show that pMN’s are specified correctly in *Gbx1*^−/−^ embryos. However, a significant decrease in the number of ISL1^+^ motor neurons in the spinal cord occurs at E14.5 in *Gbx1*^−/−^ embryos [25]. *Gbx2* lineage studies in mouse embryos have also demonstrated that a subset of ISL1^+^ ventral motor neurons derived from *Gbx2* expressing cells at E8.5 are absent in the spinal cord by E12.5 in *Gbx2*^−/−^ embryos [21]. 

**Figure 4:**
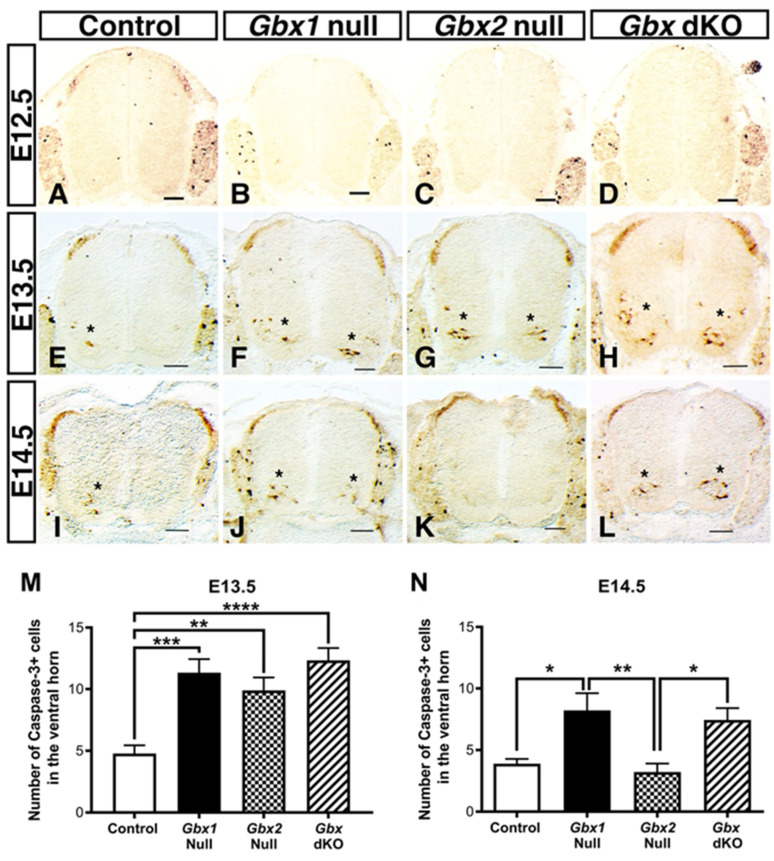
Deletion of *Gbx* genes results in apoptosis of neurons in the ventral spinal cord. Immunostaining of E12.5 (**A**–**D**), E13.5 (**E**–**H**), and E14.5 (**I**–**L**) lumbar spinal cords to detect apoptosis using caspase-3. At E12.5, no difference in the level of apoptosis is detected between any Gbx mutant when compared to the control. At E13.5, a few cells positive for caspase-3 are now observable within the ventral spinal cord of the control embryo (**E**), indicating a normal level of apoptosis that occurs under wildtype conditions. However, this level of apoptosis significantly increases within the ventral spinal cord, upon the single or double inactivation of *Gbx* genes at E13.5 in *Gbx1*^−/−^, *Gbx2*^−/−^, and *Gbx* dKO mutants (**F**–**H**,**M**). Again at E14.5, a small number of cells are caspase-3^+^ in the control embryo (**I**). The increase in apoptosis in the ventral horn is persistently observable at this stage in *Gbx1*^−/−^ and *Gbx* dKO embryos, however this phenomenon appears to terminate in *Gbx2*^−/−^ embryos, as no positive cells are detected (**J**–**L**,**N**). Black asterisks denote the presence of caspase-3 immunoreactivity. One-way ANOVA comparing the number of caspase-3^+^ cells in the ventral horn across experimental groups revealed an overall significant difference at E13.5 and E14.5 (F(3, 32) = 12.00, *p* < 0.0001 and F(3, 32) = 7.22, *p* = 0.0008, respectively). Post-hoc Tukey’s HSD revealed significant differences between all *Gbx* mutants compared to control at E13.5, but only between *Gbx1*^−/−^ embryos at E14.5 (N). Results are shown as means ± SEM. Brackets above bars in graphs denote which groups differ significantly. Samples considered statistically significant have a value of * *p* < 0.05, ** *p* < 0.01, *** *p* < 0.001, **** *p* < 0.0001. Scale bars represent 100 µm. N = 3 for each genotype, at each stage.

Therefore, to gain insight into the possible function of *Gbx* genes in cell survival, we examined spinal cord sections for immunoreactivity to caspase-3 at E12.5, E13.5, and E14.5 in *Gbx* single and dKO embryos. Caspase-3 is a cysteine-aspartic acid protease that is used to identify cells entering into the apoptotic signaling cascade [39]. We did not observe any difference in apoptotic activity in the dorsal or ventral spinal cords of control and *Gbx* mutants at E12.5 (Figure 4A–D). Interestingly, however, we observed a significant increase in activated caspase-3 in the ventral spinal cords of both *Gbx* single and dKO mutants when compared to control embryos at E13.5 (*p* < 0.01) (Figure 4E–G,M). We also noted an increase in caspase-3 activity in E13.5 *Gbx* dKO embryos. Surprisingly, the level of caspase-3 activity appears augmented in *Gbx* dKO embryos at this stage when compared to each single *Gbx* mutant (Figure 4F–H). A significant increase in apoptotic activity persists in *Gbx1*^−/−^ embryos through E14.5 (*p* = 0.0125) (Figure 4J,N). However, we did not detect any apoptotic activity above that observed in control embryos in *Gbx2* mutants at E14.5 (Figure 4I,K,N). The fact that these data show a marked increase in the amount of caspase-3^+^ cells in *Gbx* single and dKO mutants at E13.5 (Figure 4F–H,M), strongly suggests that cooperation between *Gbx1* and *Gbx2* in survival of ventral neurons occurs at this stage. 

**Figure 5:**
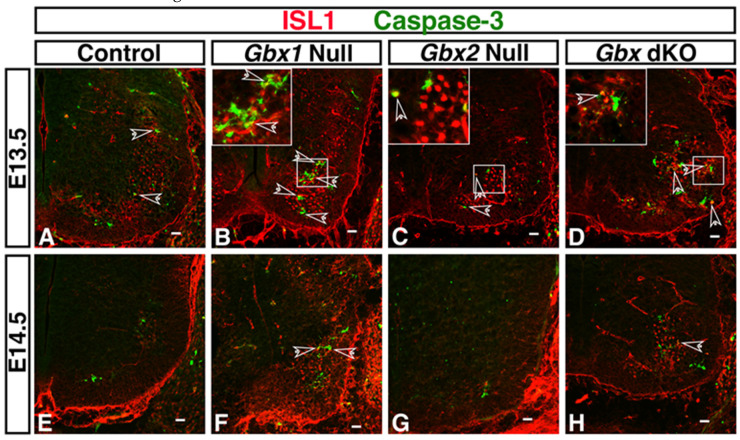
Caspase-3 colocalizes with Islet1^+^ motor neurons in the ventral spinal cord of *Gbx* mutants. Immunostaining to detect caspase-3^+^ apoptotic spinal neurons in the lumbar spinal cords of *Gbx1*^−/−^, *Gbx2*^−/−^, and *Gbx* dKO mutants at E13.5 (**A**–**D**) and E14.5 (**E**–**H**). Co-immunostaining with ISL1 antibody allows for the analysis of apoptotic neurons co-expressing the marker of spinal motor neurons. Upon analysis at E13.5, control embryos express a few cells immunopositive for caspase-3, however, none of these cells colocalize with cell bodies expressing ISl1, indicating that under normal condition at this stage, motor neurons do not undergo apoptosis. In the *Gbx* mutants however, some cells immunopositive for caspase-3 colocalize with ISL1^+^ motor neurons in the ventral spinal cord (white arrows and insets in **B**–**D**). This indicates a role for *Gbx* in survival of a subset of ISL1 populations as early as E13.5. At E14.5, apoptotic cells are detectable in the ventral spinal cord of control embryos. This level of caspase-3 immunoreactivity is persistent in *Gbx1*^−/−^ and *Gbx* dKO embryos, where a subset of apoptotic cells colocalize with ISL1^+^ motor neurons (**F** and **H**, white arrowhead). Notably, few to no cells are caspase-3^+^ in *Gbx2*^−/−^ mutant embryos. Scale bars represent 50 µm. N = 3 for each genotype, at each stage. Insets are 40× magnification of boxed regions in (**B**–**D**).

We next sought to determine if the dying cells observed in *Gbx* mutant spinal cords are indeed motor neurons. We costained transverse, lumbar spinal cord sections from *Gbx1*^−/−^, *Gbx2*^−/−^, *Gbx* dKO, and littermate control embryos by immunohistochemistry with ISL1 and caspase-3 at E13.5 (Figure 5A–D) and E14.5 (Figure 5E–H). At E13.5, very little apoptotic cell death occurs in the ventral horn of control embryos (Figure 4E and Figure 5A). Moreover, very few caspase-3^+^ cells in the ventral horn of normal control embryos colocalize with ISL1^+^ motor neurons (Figure 5A arrowheads). In contrast, we observed a modest amount of (ISL1/caspase-3) colocalization in *Gbx1*^−/−^ embryos at E13.5 (Figure 5B arrowheads, inset). Only a few of the caspase-3^+^ cells in *Gbx2*^−/−^ embryos (Figure 4G) colocalize with ISL1^+^ cells at E13.5 (Figure 5C arrowheads, inset). While to a lesser degree than at E13.5, increased caspase-3 activity is sustained in *Gbx1* and *Gbx* dKO mutants at E14.5 (Figure 4J,L). However, the majority of caspase-3 positive cells in these mutants do not appear to colocalize with ISL1^+^ motor neurons, such as that observed in controls (Figure 5E,F,H). The results of these data recapitulate our caspase-3 immunostaining analyses shown in Figure 4 and further demonstrate that loss of *Gbx* gene function results in increased apoptotic activation. Furthermore, these data reveal instances of ISL1^+^ motor neuron apoptosis in the ventral horn. Hence, these data support programmed cell death as a mechanism contributing to some motor neuron loss in *Gbx* mutants. 

## 4. Discussion

*Gbx1* and *Gbx2* are dynamically expressed during embryogenesis, and display extensive overlap in the CNS, particularly in the developing spinal cord. However, their role(s) in spinal cord development have only recently begun to be elucidated. In this study, we have focused our analysis on possible genetic and functional redundancy between them in the developing spinal cord. By analyzing *Gbx1*^−/−^, *Gbx2*^−/−^, and *Gbx1^−/−^/Gbx2*^−/−^ embryos, we demonstrate that *Gbx* function is a key regulatory component in maintenance of a subset of dorsal PAX2^+^ interneurons at E13.5 and E14.5. Further, we observed a significant amount of apoptosis in the ventral spinal cord of all *Gbx* mutants at E13.5 and in *Gbx1* at E14.5. Many of the caspase-3 immunopositive cells colocalize with ISL1^+^ motor neurons, suggesting that we have uncovered a novel mechanism through which *Gbx1* and *Gbx2* function to maintain the survival of ventral motor neurons in the developing spinal cord. These data demonstrate that *Gbx1* and *Gbx2* are essential for normal development of spinal interneurons and motor neurons in the developing spinal cord, which underlie the assembly of complex neural circuits within it.

### 4.1. Gbx1 and Gbx2 Are Required for Normal Development of Late-Born Inhibitory Interneurons in the Dorsal Spinal Cord 

The expression of *Gbx1* and *Gbx2* can be detected in the spinal cord primordium as early as E9.0 and E8.5, respectively. Differences in expression patterns of each *Gbx* gene at these early stages suggest independent regulation of distinct populations of precursor cells. However, colocalization of *Gbx* mRNA expression in the dorsal mantle zones beginning at E12.5, supports possible genetic interaction during late stages of neurogenesis. We analyzed if genetic interaction between *Gbx* transcription factors occurs, with a focus on developmental stages that their expression domains most heavily overlap within the dorsal spinal cord, E12.5–E13.5, and when *Gbx2* is significantly downregulated in normal spinal cords, E14.5. This developmental period is coincident with the generation of late-born inhibitory interneurons. Our in situ hybridization analyses at E13.5 and E14.5 show that loss-of-function in a single *Gbx* gene results in increased expression of its normal counterpart. The observed marked change in *Gbx* expression profiles within the superficial dorsal horn during the late phase of neurogenesis is intriguing, and strongly support the notion that interaction between *Gbx1* and *Gbx2* occurs at a genetic level in the dorsal spinal cord. 

We observed a striking defect in the superficial dorsal horn of *Gbx* mutant embryos, where we found clear alterations in the number of late-born PAX2^+^ cells at E13.5 and E14.5. Interestingly, studies have shown that GBX1 and GBX2 proteins are expressed in subsets of late-born PAX2^+^ cells [21,26,28,30]. Further, recent lineage studies in the dorsal spinal cord show that *Gbx2* expressing cells directly give rise to PAX2^+^ cells (Luu et al., 2011). Consistant with these studies, we observed a significant loss of PAX2^+^ expressing cells in the superficial dorsal horn in *Gbx2*^−/−^ embryos (Figure 3C,G,I,J). Additionally, our data also show that significant reduction in PAX2^+^ expressing cells occurs in *Gbx1*^−/−^ embryos (Figure 3B,F,I,J) and are consistent with evidence demonstrating a requirement for *Gbx1* in normal development of PAX2^+^ cells in the spinal cord [28]. Intriguingly, the reduction of PAX2^+^ cells is not augmented in *Gbx* dKO embryos when compared to single *Gbx* mutants (Figure 2I,J), suggesting that for this cell type, the Gbx factors do not function in a compensatory manner. One possible explanation of this paradoxical result is the traditional threshold-dependent readout model [40]. Following this model, a critical threshold level of total *Gbx* gene product is required for correct development of subsets of late-born PAX2^+^ cells. Consequently, loss of *Gbx1*, *Gbx2*, or both would result in similar cell number defects. This concept is supported by recent studies showing that patterning of distinct tissues relies on how transcription factor concentration is perceived by the underlying genetic network [41]. In addition, results from our recent studies demonstrate varying threshold requirements for *Gbx2* gene product in different regions of the developing anterior hindbrain [19]. While it remains to be determined if GBX1 and GBX2 proteins are colocalized in the same subpopulation(s) of PAX2^+^ cells, these data reveal remarkable parallels between *Gbx1* and *Gbx2* in development of a dorsal inhibitory interneuron population. 

Reduced cell proliferation and programmed cell death are two common mechanisms that underlie loss of spinal neurons in normally developing embryos. Although we observe substantial loss of PAX2^+^ cells throughout the superficial dorsal horn at E13.5 and E14.5 (Figure 4), our data shows very little apoptotic cell death, as indicated by caspase-3 activity, occurring in the dorsal horn of *Gbx* single or dKO mutants. Importantly, this finding is consistent with prior studies demonstrating that dorsal spinal interneuron apoptosis normally begins after E17 in mice, and occurs primarily after postnatal day (P) 0 [42,43]. Therefore, these data would support that *Gbx* genes could function in regulating the proliferative status of dorsal PAX2^+^ inhibitory interneurons. This view is supported by results from our previous studies in mice and zebrafish demonstrating that *Gbx2* does indeed function in maintaining the proliferative status of neural progenitors in the cerebellar anlage and transient anterior hindbrain structures, rhombomeres (r) 2, r3, and r5 [19,24]. This notion is also consistent with a recent study proposing *Gbx2* to maintain the proliferative status of dorsal progenitors [21]. While the present study does not exhaustively address the proliferative status of dorsal inhibitory interneurons, our data do reveal a prominent decrease in the amount of PAX2^+^ cells in the deep dorsal horn, where dI4 and dIL progenitors reside, in *Gbx* single and dKO mutants at E13.5. It will be interesting in future studies to see if regulation of spinal cord proliferation by *Gbx* acts by impacting the expression of morphogenic signaling molecules such as FGFs and Wnts as shown in the anterior hindbrain [19,27].

### 4.2. Gbx Family Members Act to Maintain Ventral Motor Neuron Populations through a Shared Molecular Mechanism 

We also observed phenotypic parallels in the ventral spinal cord of *Gbx* mutants. In striking contrast to the dorsal spinal cord, we observed an abundance of caspase-3 activity in the ventral horns of all *Gbx* mutant spinal cords at E13.5 (Figure 4F–H,M). *Gbx1*^−/−^ embryos also display increased apoptosis at E14.5 (Figure 4J–L,N). Notably, we did observe some ventral apoptosis in normal control embryos at these stages. Importantly, our observation of ventral apoptosis in control embryos is consistent with previous results indicating that spinal interneuron apoptosis proceeds in a ventral-to-dorsal temporal gradient and correlates with the first wave of synaptogenesis in the spinal cord between E14 and 17 [43,44,45]. Our recent analysis of *Gbx1*, demonstrate that loss of *Gbx1* function results in a significant reduction in ventral ISL1^+^ motor neurons beginning at E14.5, which persists through P5. Similarly, *Gbx2* fate mapping studies in mice have shown that motor neurons derived from the *Gbx2* lineage are lost in *Gbx2* null embryos beginning at E12.5 [21]. However, the mechanism(s) underlying the loss of motor neurons in the *Gbx* mutant spinal cords was not determined in either study. In this study, we show increased caspase-3 activity in *Gbx* mutant spinal cords at E13.5 which colocalizes with ISL1^+^ motor neurons. Hence, these data provide first evidence supporting that *Gbx* gene function maintains ISL1^+^ motor neuron survival by preventing apoptotic cell death. 

Whether, *Gbx1* and *Gbx2* play direct cell-autonomous or non-cell-autonomous roles in ventral motor neuron survival remains unresolved. The notion that *Gbx* genes may have non-cell-autonomous roles in motor neuron survival is intriguing. Indeed, this has been previously postulated for *gbx2* function in r2–r3 of zebrafish hindbrain where little or no *gbx2* is expressed [24]. Similarly, in situ hybridization studies in several species demonstrating that *Gbx2* mRNA is barely detectable ventral of the EN1^+^, V1 interneuron domain in the ventral spinal cord, also provide support for non-cell-autonomous function in motor neuron survival [29,46,47]. Furthermore, several HD-containing transcription factors, En1, En2, Pax6, and Otx2 have been shown to function as secreted factors in shaping neural circuits [48,49,50]. Similar to *Gbx2*, our recent analysis of *Gbx1*^−/−^ embryos also supports the possibility of non-cell-autonomous contributions in motor neuron survival by *Gbx* transcription factors. We have shown that projection of proprioceptive afferents into the ventral spinal cord to establish connections with ISL1^+^ motor neurons terminates prematurely in the intermediate zone of *Gbx1*^−/−^ spinal cords [25]. Therefore, the observed increase in apoptotic motor neurons in *Gbx1*^−/−^ spinal cords may be due to loss of functional synapses and not cell-autonomous requirements. 

We show here that heavy caspase-3 activity occurs in the spinal cords of both *Gbx* single mutants at E13.5, and that activity is increased in *Gbx* dKO embryos at this stage. Surprisingly, however, while apoptosis persists in *Gbx1* single and dKO mutants at E14.5, it is severely diminished in *Gbx2*^−/−^ spinal cords at this stage (Figure 4). One possible interpretation of these results is that *Gbx1* and *Gbx2* function to regulate distinct subpopulations of ISL1^+^ motor neurons undergoing apoptosis at E13.5. While at E14.5 only those ISL1^+^ motor neurons being impacted by *Gbx1* continue to undergo apoptosis. Interestingly, this view is consistent with recent fate-mapping studies suggesting that the *Gbx2* lineage contributes to ventromedial ISL1/2^+^ motor neurons that constitute the medial motor column (MMC). Whereas, our recent functional analysis strongly suggest, that motor neurons constituting the lateral motor column (LMC) are affected by the *Gbx1* null mutation [21,25,51]. Furthermore, it is noteworthy that loss of motor neurons occurs in *Gbx1*^−/−^ and *Gbx2*^−/−^ mutants, suggesting that neither gene functionally compensates for the loss of a family member in distinct ISL1^+^ motor neuron subpopulations. A thorough investigation through gene replacement analysis will provide more insight into this possibility. While these data do not support a potential for functional redundancy between *Gbx* genes in ventral ISL1^+^ motor neuron development, they do however, demonstrate *Gbx* genes have a shared role in motor neuron survival by preventing apoptosis. 

## 5. Conclusions

We have shown that the dorsal and ventral regions of the spinal cord are affected in a remarkably parallel manner in *Gbx1*^−/−^ and *Gbx2*^−/−^ mouse embryos. Our results provide evidence that *Gbx1* and *Gbx2* function similarly, but not redundantly, within the cellular subtypes that they together act to generate and maintain. Our observations of increased *Gbx1* and *Gbx2* mRNA expression in their null counterparts, a decrease in PAX2^+^ dorsal interneurons, as well as increased apoptosis in a subset of ventral ISL1^+^ motor neurons in *Gbx* single and dKO mutants demonstrate that we have uncovered novel mechanisms through which *Gbx* genes regulate development and maintain the survival of PAX2^+^ dorsal interneurons and ventral ISL1^+^ motor neurons, respectively, in the developing spinal cord.
